# Determinants of quality of life among Malaysian cancer patients: a cross-sectional study

**DOI:** 10.1186/s12955-018-0989-5

**Published:** 2018-08-13

**Authors:** Mehrnoosh Akhtari-Zavare, Sherina Mohd-Sidik, Ummavathy Periasamy, Lekhraj Rampal, Siti Irma Fadhilah, Rozi Mahmud

**Affiliations:** 1 Department of Public Health, Tehran Medical Sciences Branch, Islamic Azad University, Tehran, Iran; 2 Cancer Resource & Education Center, Universiti Putra Malaysia, 43400 Serdang, Selangor Malaysia; 3 Department of Psychiatry, Faculty of Medicine & Health Sciences, Universiti Putra Malaysia, 43400 Serdang, Selangor Malaysia; 4 Hospital Tuanku Jaafar, Seremban, Negeri Symbilan Malaysia; 5 Department of Community Health, Faculty of Medicine & Health Sciences, Universiti Putra Malaysia, 43400 Serdang, Selangor Malaysia

**Keywords:** Quality of life, Cancer patients, Psychological effects, Malaysia

## Abstract

**Background:**

Cancer is a serious public health problem not only in Malaysia, also worldwide. The aim of this study was to determine the determinants of quality of life (QOL) among cancer patients in Peninsular Malaysia.

**Methods:**

A cross sectional study was conducted among 2120 cancer patients in Peninsular Malaysia, between April 2016 to January 2017. All cancer patients aged 18 years old and above, Malaysian citizens and undergoing cancer treatment at government hospitals were approached to participate in this study and requested to complete a set of validated questionnaires. Inferential statistical tests such as t-test and one-way ANOVA were used to determine the differences between demographic variables, physical effects, clinical factors, psychological effects and self-esteem with the quality of life of cancer patients. Predictor(s) of quality of life were determined by using Multivariate linear regression models.

**Result:**

A total 1620 out of 2120 cancer patients participated in this study, giving a response rate of 92%. The majority of cancer patients were female 922 (56.9%), Malays 1031 (63.6%), Muslim 1031 (63.6%), received chemotherapy treatment 1483 (91.5%). Overall, 1138 (70.2%) of the patients had depression and 1500 (92.6%) had anxiety. Statistically significant associations were found between QOL and clinical factors, physical side effects of cancer, psychological effects and self-esteem (*p* < 0.05). However, among socio-demographics only age, race, religion, working status were significantly associated with QOL. Based on the multivariate regression analysis, the main predictors of QOL among cancer patients in Malaysia were age, self-esteem as positive predictors, and Indian race, nausea, fatigue, hair loss, bleeding as negative predictors.

**Conclusion:**

The findings of this study provide a scientific basis to develop a comprehensive program for improving quality of life of cancer patients in Malaysia.

## Background

Cancer is one of the leading causes of morbidity and mortality worldwide. In 2012, there were approximately 14.1 million new cases, 8.2 million cancer deaths and 32.6 million people living with cancer worldwide [1]. In Malaysia, cancer is one of the major health problems and a 3rd leading cause of premature death in this country [2, 3]. Based on the National Cancer Registry [4], in the period of 2007–2011, 103,507 new cancer cases and 64,275 cancer deaths were reported in Malaysia, which had increased fivefold from 2003 (21,464 cases of cancer) [4]. However, the survival rate of cancer is also increasing in Malaysia, therefore improving quality of life (QOL) among cancer survivors in Malaysia and the world should have significant public health implication [2, 5]. QOL is a multidimensional, multifaceted measure which refers to an individual’s general wellbeing, including mental, emotional, social, and physical aspects of the individual’s life [6]. Recently in oncology medicine, QOL was viewed as a primary end point measure to assess the efficacy of treatment among patients [7] and reflects patients’ opinion about the effects of cancer diagnosis and treatment on daily living [8, 9]. Several studies reported improving and better QOL among cancer patients associated with longer survival rates [5, 6]. Numerous studies reported that level of education [10], type of treatment [11], marital status [12], monthly income [13], age at cancer diagnosis [14], cancer type [15], cycle of cancer treatment [15], anxiety [16, 17], and depression [16, 17] are associated with QOL among cancer patient survivors. However, most of these studies are conducted in western countries with different life styles and cultures. These results are not applicable for developing countries such as Malaysia which has a different lifestyle and culture from Western societies.

The present study is part of a research program designed to assess the effectiveness of a chemotherapy counselling module to improve QOL among cancer patients in Peninsular Malaysia. The aim of this study was to assess the determinants of QOL among cancer patients in Peninsular Malaysia.

## Methods

### Study design

A cross sectional study was conducted between April 2016 to January 2017 and patients were recruited from 10 selected government hospital with oncology facilities serving the whole of Peninsular Malaysia. The whole of Malaysia is made up of West Malaysia (also known as Peninsular Malaysia) and East Malaysia (consisting of Sabah and Sarawak). The protocol of study was approved by Medical Ethics Committee (MREC), Ministry of Health, Malaysia, National Medical Research Registry (NMRR), Universiti Pura Malaysia Ethics Committee (JKEUPM) as well as the Hospital Directors of the selected government hospitals. Written consent was obtained from all patients before they were enrolled in the study.

### Recruitment and eligibility screening

All cancer patients (stage 1–4) aged 18 years old and above, Malaysian citizens, who were undergoing first and second cycles of chemotherapy treatment in government hospitals with oncology facilities in Malaysia and who were able to complete the questionnaires independently or with help were recruited. Patients with diagnosed psychiatric disorders who were on treatment and follow-up, and those with severe communication problems such as speech or hearing difficulties and those who were undergoing the third cycle of chemotherapy onwards were excluded from the present study.

The eligible participants were informed about the purpose of study, date and place of study by the pharmacists (site investigators) of the selected hospitals and he/she served as a contact person for each selected hospital and was responsible for the recruitment of patients. Patient recruitment occurred on a daily basis where consecutive patients attending participating hospitals for chemotherapy were invited to participate. The recruitment process continued until a total of 212 patients were obtained at each selected hospital.

### Sampling method

**Fig. 1:**
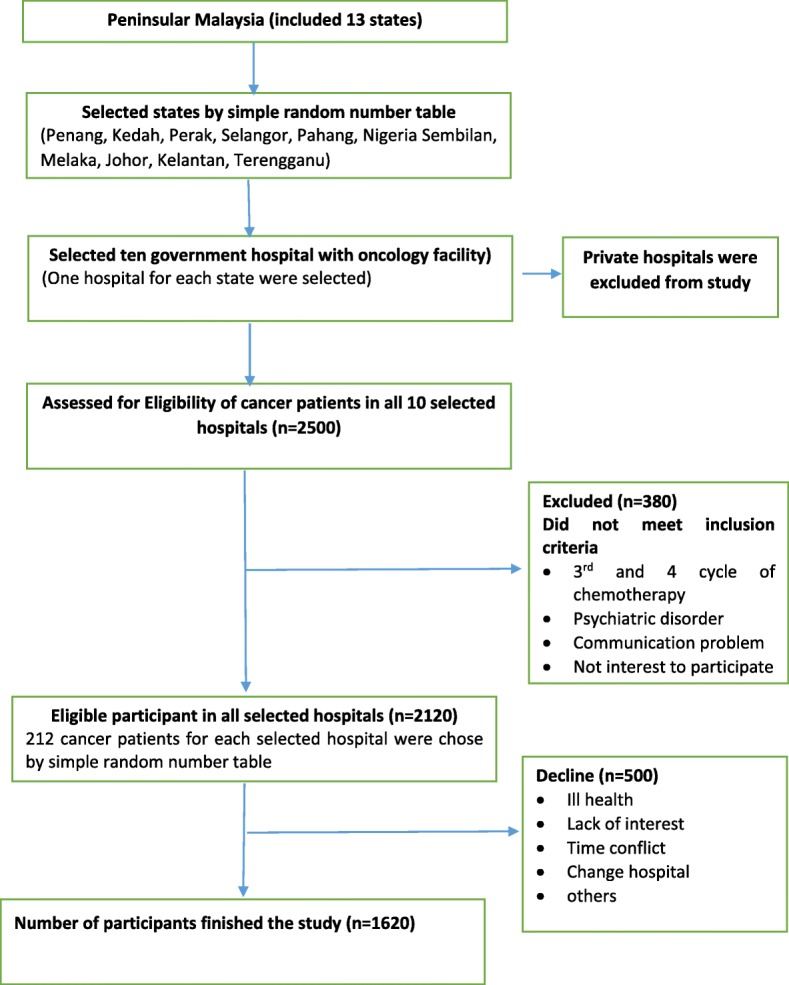
**Flow chart of sampling method**. The above figure shows the sampling method of this research. The sample frame for government hospitals with oncology facilities was obtained from Ministry of Health, Malaysia. Also, the sampling frame for cancer patients was obtained from the Cytotoxic Drug Reconstitution (CDR) pharmacy department of each selected hospital

The multistage random sampling method was used for selecting participants. First, 10 states out of 13 states in Peninsular Malaysia were selected randomly. Due to limited resources for investigators to undertake traveling to East Malaysia, Sabah and Sarawak were not included in this study. Second, a list of government hospitals with oncology facilities in each state was obtained from the Ministry of Health, Malaysia; where one government hospital was randomly chosen from each state by using the random number table (a total of 10 hospitals were selected). Finally, in each selected hospital, 212 cancer patients were randomly selected from the list of all eligible cancer patients which was obtained from the cytotoxic drug reconstitution (CDR), Pharmacy Department of each selected hospital. The randomly selected participants followed a standardized set of criteria as stated in the recruitment section. All cancer patients were registered in the CDR of each selected hospital before undergoing their treatment. In this study, private hospitals in Peninsular Malaysia were not chosen because they did not have similar standardized based operative procedures as the government hospitals. The fellow chart of sampling method is shown as in Fig. 1.

### Sample size

The Rosner’ formula (*n* = [zα√pq (1 + 1/k) + zβ√p1q1 + p2q2/k]2/Δ2) was used for sample size estimation [18]. In order to achieve 80% power (2-sided alpha *p* = 0.05) to detect a group difference of 8% [19] with 20% attrition, 212 cancer patients in each selected hospital were required. The final sample size for all 10 hospitals was 2120 cancer patients. Among those who initially agreed to participate in this study 500 dropped out because of a variety of reasons (no longer willing to participate, moving to another hospital, time conflict, and not feeling well). As a result of this, 1620 were enrolled in the present study, giving a response rate of 92%.

### Procedures of data collection

Data were collected by a group of trained research assistants (RAs). Before collecting the data, the purpose of the study and the details about the study were provided by an information sheet for each selected participant. Written consent was obtained from the participants before the questionnaires were administered. Each questionnaire was checked for completeness after being answered by the participants. Souvenirs were given to each participant and they were all thanked for their participation. In the current study, investigators collected sufficient clinical data required for the research from the patients via the patient’s own record books. The investigators could not have access to the hospital medical records due to confidentiality and ethical issues.

### Instruments

The questionnaires used in this study included questions on socio-demographic information, clinical factors, physical side effects, anxiety, depression, self-esteem and QOL. Data were collected via self-administered Malay questionnaires which had been validated into the Malay language (the National language of Malaysia) from the original instruments [20–23].

### Socio-demographic characteristics

Items on socio-demographic characteristics included age, gender, race, religion, level of education, marital status, family income, working status, and family history of cancer.

### Clinical factors

This part consist of cancer treatment, cancer stage, type of cancer, type of cancer treatment and pain due to cancer.

### Common terminology criteria for adverse events (CTCAE)

The CTCAE is a self-report questionnaire used for assessing physical side effects of cancer treatment. It provides information about vomiting, nausea, anorexia, fatigue, hair loss, bleeding and infection due to cancer treatment. The validity and reliability of questionnaire was checked among cancer patients in Selangor, Malaysia [20].

### Patient health Questionnaire-9 (PHQ-9)

The validated Malay version of the PHQ-9 with good sensitivity (87%, 95% CI 71% to 95%) and specificity of (82%, 95%CI 74% to 88%) was used to assess the presence of depression in this study [21]. The PHQ-9 is a self-report instrument which assesses the presence of depression based on the DSM-IV criteria [24]. It consists of 9 items, each item scored from 0 (not at all) to 3(nearly every day), with score ranges from 0 to 27. In this study a threshold score of 10 or above on the PHQ-9 was considered as the presence of depression among participants. A cut off point of 10 and above was used as it has the optimum level of specificity and sensitivity [21].

### Generalized anxiety disorder-7(GAD-7) questionnaire

The GAD-7 is a self-report instrument for assess generalized anxiety disorder [25]. The GAD-7 has seven items with scores range from 0 to 27 and each item was scored from 0 (not at all) to 3 (nearly every day). In this study, the presence of anxiety was determined by using a cut-off point of 8 and above on the GAD-7 [22]. The validated Malay version of GAD-7 which was found to have good specificity (94%, 95% CI 88–97) and sensitivity (96%, 95% CI 61–87) was used in this study [22].

### Rosenberg self-esteem scale (RSES)

The Rosenberg Self-Esteem Scale (RSES) was developed and validated by Morris Rosenberg to measure global self-esteem [26]. The validated Malay version of Rosenberg was used in this study [24]. It consists of 10 questions related to self-esteem using a four-point Likert scale ranging from “strongly agree” (1) to “strongly disagree” (4).

### WHO quality of life-BREF (WHOQOL-BREF)

The validated Malay version of the WHOQOL-BREF was used in this study with acceptable intra-class correlation coefficient (ICC) value from 0.79–0.88 [27]. The WHOQOL-BREF consists of 26 items comprising four subscales: (a) physical (7 items), (b) psychological (6 items), (c) environment (8 items) and (d) social relationships (3 items). Response categories are associated with a five level scale from “very poor” (1) to “very good” (5) and higher scores indicate a better QOL, also, there are no cut–off points for any domains.

### Data analysis

We used the Statistical Package for Social Science (SPSS) version 22.0 for data analysis and the demographic data and outcome measures of the study variables described by descriptive statistics such as: frequency, percentage, mean and standard deviation. Normality tests were done with the Shapiro-Wilk test and all of the quantitative data were found to be normally distributed. Regarding scoring for each of the four domains of the WHOQOL-BREF, the scores (0–100 scale) were obtained after linear transformation of a combination of n variables coded 1 to 5 (Likert scale) [28]. Parametric tests such as t-test and one-way ANOVA were employed to determine the relationships between QOL and independent variables (socio-demographic, clinical factors, physical side effects and psychological effects), while Pearson correlation coefficient was conducted between QOL and self-esteem. Post hoc analysis was used to find where the significant differences actually occurred in selected variables (variables which were significant) at a new *p*-value of ≤0.005 after Bonferroni adjustment. Multivariate regression regression analysis was employed to determine the predictors of QOL. The enter method was used. Significant variables (*p* < 0.05) from Pearson correlation coefficient, t-test and one-way ANOVA were inserted into the logistic regression model, and those which were not significant (*p* > 0.05) were removed from the model. The level of statistical significance was set at α < 0.05.

## Result

### Participants

Out of 2120, the minimum sample size of 1620 patients were enrolled into the present study (92% response rate). Table 1 presents the distribution of cancer patients in terms of socio-demographics, clinical factors, physical side effects, psychological effects and self-esteem. The majority of cancer patients were female 922(56.9%), Malays 1031(63.6%), Muslim 1031(63.6%), married 1115(68.8%), with education level of diploma and below 934(57.7%). Family history of cancer was reported by 750 (46.3%) of the respondents and more than half of the study participants 876(54.1%) had pain due to cancer. With regards to types of cancer 523(32.3%) and 450 (27.8%) had breast and colorectal cancer, respectively. All participants received chemotherapy, where more than two-thirds of them 1483(91.5%) were on chemotherapy treatment alone. About 61% (*n* = 989) were undergoing their first cycle of chemotherapy, while 609(37.6%) were already in stage IV of cancer. Regarding treatment side effect, majority of respondent had more than three treatment side effect 1275 (78.7%) and only 107 (6.6%) had no treatment side effect. In detail of treatment side effect a greater percentage of respondents had vomiting 1145(70.7%), nausea 1033(63.7%), anorexia 1237(76.4%), fatigue 1418(87.5%) and hair loss 1394(86.0%). Also, approximately 1138 (70.2%) of the patients had depression and 1500 (92.6%) had anxiety. Table 1 summarizes characteristics of respondent.

**Table 1:** Characteristics of cancer patients in Malaysia (*n* = 1620)

Socio-demographic Characteristics	No	%
Age (year)		
< 45	213	13.1
45–54	278	17.2
55–64	513	31.7
> 65	616	38.0
Mean ± SD	55.9 ± 13.34	
Range	17–89	
Gender		
Male	698	43.1
Female	922	56.9
Race		
Malays	1031	63.6
Chinese	380	23.5
Indian	196	12.1
Other	13	0.8
Religious		
Muslim	1031	63.6
Buddhist	380	23.5
Hindu	196	12.1
Christian	13	0.8
Marital status		
Married	1115	68.8
Single	110	6.8
Divorce/widow	395	24.4
Education level		
No formal education	367	22.7
Below diploma	934	57.6
University level	319	19.7
Family Income (MYR)©		
No income	591	36.5
< 1500 MYR	293	18.1
1501–3500 MYR	411	25.4
> 3501 MYR	325	20.0
Working status		
Yes	703	43.4
No	591	36.5
Retired	326	20.1
Family history of cancer		
Yes	750	46.3
No	870	53.7
Type of Cancer		
Breast	523	32.3
Ovarian	62	3.8
Cervical	143	8.8
Colorectal	450	27.8
Lymphoma	102	6.3
Stomach	161	9.9
Others	179	11.1
Cancer stage		
I	161	9.9
II	250	15.4
III	600	37.1
IV	609	37.6
Type of cancer treatment		
Chemotherapy	1483	91.5
Chemotherapy & radiation	137	8.5
Cycle of cancer treatment		
1st cycle	989	61.0
2nd cycle	631	39.0
Pain due to cancer		
Yes	876	54.1
No	744	45.9
Treatment Side Effect	No	%
Treatment side effect		
No side effect	107	6.6
One side effect	62	3.8
Two side effect	176	10.9
≥ Three side effect	1275	78.7
Vomiting		
Yes	1145	70.7
No	475	29.3
Nausea		
Yes	1033	63.7
No	587	36.3
Anorexia		
Yes	1237	76.4
No	383	23.6
Fatigue		
Yes	1418	87.5
No	202	12.5
Hair loss		
Yes	1394	86.0
No	226	14.0
Bleeding		
Yes	688	42.4
No	932	57.6
Infection		
Yes	1175	72.5
No	445	27.5
Psychological Factor	No	%
Depression		
Yes (PHQ-9 ≥ 10)	1138	70.2
No (PHQ-9 < 10)	482	29.8
Anxiety		
Yes (GAD-7 ≥ 8)	1500	92.6
No (GAD-7 < 8)	120	7.4
Self-esteem		
Mean ± SD	24.59 ± 7.00	
Range	13–37	

© 1USD = 4 MYR, *SD* standard deviation

### Factors associated with quality of life

#### Socio-demographics

The associations between socio-demographic characteristics and total QOL are reported in Table 2. Age (*p* < 0.001), race (*p* < 0.001), religion (*p* < 0.000) and working status (*p* < 0.001) were significantly associated with all domains of QOL in the bivariate analysis. Single women had significant higher scores in the environment domain of QOL (*p* < 0.04); while cancer patients with education level of diploma and less had higher scores on physical health and lower scores on psychological health (*p* < 0.03). Not significant differences was found between gender and total QOL and each domains of QOL (Table 2).

**Table 2:** Quality of life measures by socio-demographic characteristics in cancer patients in Malaysia (*n* = 1620)

Socio-demographic	Physical health	Psychological health	Social relationships	Environment
	Mean ± SD	Mean ± SD	Mean ± SD	Mean ± SD
Age (year)				
< 45	65.43 ± 16.50	58.88 ± 21.28	58.13 ± 24.27	64.86 ± 17.10
45–54	66.72 ± 18.91	67.13 ± 20.24	61.78 ± 24.76	66.42 ± 19.43
55–64	65.65 ± 20.30	59.54 ± 18.16	58.39 ± 23.17	63.83 ± 20.09
> 65	62.06 ± 21.54	58.51 ± 18.21	57.06 ± 23.55	60.82 ± 22.11
Statistics	*F* = 4.86, *p* < 0.002*	*F* = 14.47, *p* < 0.001*	*F* = 2.54, *p* < 0.06	*F* = 5.72, *p* < 0.001*
Gender				
Male	63.85 ± 20.73	61.33 ± 18.32	58.37 ± 23.38	63.22 ± 20.82
Female	64.88 ± 19.74	59.63 ± 19.85	58.48 ± 24.07	63.30 ± 20.28
Statistics	*t* = −1.01, *p* < 0.31	*t* = 1.76, *p* < 0.79	*t* = −0.09, *p* < 0.92	*t* = −0.07, *p* < 0.94
Race				
Malay	65.97 ± 18.22	61.29 ± 18.48	60.03 ± 25.25	64.72 ± 20.60
Chinese	66.56 ± 20.75	62.28 ± 18.88	60.27 ± 22.75	65.22 ± 18.44
Indian	52.39 ± 24.60	52.31 ± 21.92	46.81 ± 22.86	50.17 ± 25.64
Other	62.92 ± 16.70	52.69 ± 11.60	41.07 ± 16.74	62.92 ± 16.70
Statistics	*F* = 28.02, *p* < 0.001*	*F* = 14.57, *p* < 0.001*	*F* = 21.33, *p* < 0.001*	*F* = 32.15, *p* < 0.001*
Religion				
Muslim	65.99 ± 18.22	61.30 ± 18.48	59.92 ± 25.30	64.66 ± 20.60
Buddhist	66.50 ± 20.75	62.23 ± 18.88	60.31 ± 22.73	65.24 ± 18.43
Hindu	52.39 ± 24.60	52.31 ± 21.92	46.81 ± 22.86	50.17 ± 25.64
Christian	62.92 ± 16.70	52.69 ± 11.60	41.07 ± 16.74	62.92 ± 16.70
Statistics	*F* = 28.00, *p* < 0.000*	*F* = 14.54, *p* < 0.000*	*F* = 21.35, *p* < 0.000*	*F* = 32.17, *p* < 0.000*
Marital status				
Married	64.83 ± 19.77	60.71 ± 19.10	57.70 ± 23.05	63.81 ± 20.19
Single	65.69 ± 16.65	59.55 ± 22.36	57.57 ± 26.61	65.47 ± 16.42
Divorce/widow	62.97 ± 22.08	59.60 ± 18.61	60.74 ± 24.83	61.11 ± 22.22
Statistics	*F* = 1.46, *p* < 0.23	*F* = 0.59, *p* < 0.55	*F* = 2.45, *p* < 0.08	*F* = 3.21, *p* < 0.051
Education level				
No formal education	61.33 ± 19.25	58.25 ± 17.33	59.65 ± 22.13	60.03 ± 19.76
Diploma & Less	65.44 ± 20.92	61.31 ± 19.37	58.66 ± 23.88	64.25 ± 21.23
University level	65.08 ± 18.63	60.02 ± 20.64	56.36 ± 25.20	64.11 ± 18.80
Statistics	*F* = 5.69, *p* < 0.003*	*F* = 3.40, *p* < 0.033*	*F* = 1.74, *p* < 0.17	*F* = 5.95, *p* < 0.003*
Family Income (MYR)©				
No income	65.13 ± 19.30	61.52 ± 18.38	61.37 ± 22.00	64.12 ± 19.20
< 1500 MYR	65.02 ± 18.00	59.01 ± 18.24	54.13 ± 24.70	63.84 ± 18.19
1501–3500 MYR	63.06 ± 22.35	61.52 ± 19.95	60.04 ± 23.28	62.00 ± 23.42
> 3501 MYR	64.40 ± 20.68	58.02 ± 20.38	54.93 ± 25.66	62.80 ± 20.83
Statistics	*F* = 0.95, *p* < 0.41	*F* = 3.32, *p* < 0.019*	*F* = 9.33, *p* < 0.001*	*F* = 0.99, *p* < 0.39
Working status				
Yes	66.41 ± 20.99	61.89 ± 19.89	60.34 ± 24.47	65.18 ± 21.43
No	65.16 ± 19.31	61.59 ± 18.38	61.37 ± 22.00	64.13 ± 19.21
Retired	58.87 ± 18.92	54.85 ± 18.26	49.00 ± 23.03	57.57 ± 19.79
Statistics	*F* = 16.44, *p* < 0.001*	*F* = 17.16, *p* < 0.001*	*F* = 33.74, *p* < 0.001*	*F* = 16.48, *p* < 0.001*
Family history of cancer				
Yes	64.78 ± 19.82	61.61 ± 17.49	59.55 ± 23.37	63.60 ± 20.00
No	64.14 ± 20.48	59.29 ± 20.54	57.47 ± 24.09	62.97 ± 20.94
Statistics	*t* = 0.64, *p* < 0.52	*t* = 2.43, *p* < 0.06	*t* = 1.75, *p* < 0.08	*t* = 0.61, *p* < 0.53
Total	64.44 ± 20.18	60.36 ± 26	58.43 ± 23.77	63.26 ± 20.51

© 1USD = 4 MYR; *Significant at *p* < 0.05

While some results were statistically significant, the small differences might not be meaningful. For example; although the differences were statistically significant for age and physical health the difference essentially comes from the patients aged 65 and older but the absolute difference is only 3.5.

The highest quality of life score was found in physical health (64.44 ± 20.18), followed by environment (63.26 ± 20.51), psychological health (60.36 ± 26) and social relationship (58.43 ± 23.77).

#### Physical side effect & clinical factors

Association between QOL and clinical factors and physical side effects of cancer treatment among cancer patients in Malaysia are shown in Table 3. Cancer patients in stage 1 of cancer and under chemotherapy treatment who do not have pain due to cancer reported significantly higher scores each domains of QOL, except for type of treatment and social relationship (*p* < 0.73). Regarding the physical side effect of cancer treatment, there were statistically significant differences between vomiting (*p* < 0.001), nausea (*p* < 0.001), hair loss (*p* < 0.001), fatigue (*p* < 0.001), anorexia (*p* < 0.001), bleeding (*p* < 0.001), infection and each domains of QOL among cancer patients (Table 3).

**Table 3:** Quality of life measures by clinical factor and treatment side effect in cancer among cancer patients in Malaysia (*n* = 1620)

Clinical factor	Physical health	Psychological health	Social relationships	Environment
	Mean ± SD	Mean ± SD	Mean ± SD	Mean ± SD
Type of Cancer				
Breast	65.30 ± 19.13	58.35 ± 19.56	57.90 ± 24.65	63.74 ± 18.86
Ovarian	75.03 ± 23.19	77.00 ± 14.21	72.70 ± 22.21	72.22 ± 23.93
Cervical	68.43 ± 13.94	59.76 ± 15.32	67.62 ± 17.64	67.86 ± 14.38
Colorectal	61.98 ± 20.17	60.05 ± 17.64	58.43 ± 23.23	61.28 ± 20.88
Lymphoma	60.45 ± 19.56	55.66 ± 18.88	56.39 ± 20.86	58.60 ± 18.51
Stomach	60.67 ± 22.25	60.47 ± 24.27	49.43 ± 22.36	59.30 ± 24.92
Others	66.89 ± 22.45	64.36 ± 18.06	56.96 ± 25.81	66.32 ± 21.83
Statistics	*F* = 7.25, *p* < 0.00*	*F* = 11.47, *p* < 0.00*	*F* = 11.87, *p* < 0.00*	*F* = 6.58, *p* < 0.00*
Cancer stage				
I	67.01 ± 20.46	67.08 ± 22.53	62.08 ± 27.54	67.08 ± 20.06
II	63.04 ± 18.33	59.93 ± 18.74	56.59 ± 22.04	62.18 ± 19.40
III	67.92 ± 18.61	60.16 ± 19.06	61.92 ± 23.49	66.41 ± 18.95
IV	60.90 ± 21.63	57.57 ± 17.50	54.79 ± 23.07	59.61 ± 21.88
Statistics	*F* = 13.80, *p* < 0.001*	*F* = 8.56, *p* < 0.001*	*F* = 11.07, *p* < 0.001*	*F* = 13.52, *p* < 0.001*
Type of cancer treatment				
Chemotherapy	64.84 ± 19.91	60.91 ± 18.97	58.49 ± 23.58	63.63 ± 20.22
Chemotherapy & radiation	60.04 ± 22.43	54.43 ± 20.88	57.78 ± 25.80	59.34 ± 23.13
Statistics	*t* = 2.67, *p* < 0.008*	*t* = 3.79, *p* < 0.001*	*t* = 0.33, *p* < 0.73	*t* = 2.34, *p* < 0.01*
Cycle of cancer treatment				
1st cycle	68.43 ± 18.21	64.38 ± 19.73	64.43 ± 22.92	67.54 ± 18.65
2nd cycle	61.89 ± 20.95	57.80 ± 18.77	54.60 ± 23.52	60.54 ± 21.17
Statistic	*t* = 6.44, *p* < 0.001*	*t* = 6.80, *p* < 0.001*	*t* = 8.28, *p* < 0.001*	*t* = 6.79, *p* < 0.001*
Pain due to cancer				
Yes	60.74 ± 21.79	57.08 ± 17.44	56.08 ± 23.20	59.13 ± 22.04
No	67.58 ± 18.12	63.15 ± 20.20	60.43 ± 24.08	66.78 ± 18.41
Statistic	*t* = −6.88, *p* < 0.001*	*t* = − 6.41, *p* < 0.001*	*t* = − 3.68, *p* < 0.001*	*t* = − 7.61, *p* < 0.001*
TREATMENT SIDE EFFECT				
Treatment side effect				
No side effect	81.97 ± 14.68	76.40 ± 8.68	79.97 ± 8.60	82.98 ± 12.24
One side effect	82.75 ± 10.65	79.74 ± 10.52	83.09 ± 7.61	79.98 ± 10.40
Two side effect	81.43 ± 10.70	81.35 ± 6.22	81.81 ± 9.71	79.79 ± 8.78
≥ Three side effect	59.73 ± 19.34	55.18 ± 18.02	52.20 ± 22.66	58.52 ± 19.99
Statistic	*F* = 135.82, *p* < 0.001*	*F* = 201.08, *p* < 0.001*	*F* = 183.96, *p* < 0.001*	*F* = 133.99, *p* < 0.001*
Vomiting				
Yes	57.45 ± 18.89	52.43 ± 16.60	49.44 ± 21.47	56.23 ± 19.61
No	81.28 ± 11.25	79.49 ± 8.82	80.11 ± 12.31	80.21 ± 10.05
Statistic	*t* = −25.66, *p* < 0.001*	*t* = − 33.58, *p* < 0.001*	*t* = − 29.19, *p* < 0.001*	*t* = − 25.29, *p* < 0.001*
Nausea				
Yes	54.07 ± 16.95	49.55 ± 14.87	47.79 ± 20.55	53.11 ± 18.07
No	82.68 ± 9.81	79.39 ± 7.88	77.17 ± 16.32	81.13 ± 9.23
Statistic	*t* = −37.46, *p* < 0.001*	*t* = − 45.13, *p* < 0.001*	*t* = − 29.71, *p* < 0.001*	*t* = − 35.04, *p* < 0.001*
Anorexia				
Yes	59.37 ± 19.43	54.72 ± 17.86	51.68 ± 22.66	58.10 ± 20.05
No	80.82 ± 12.29	78.59 ± 9.83	80.24 ± 10.46	79.94 ± 10.78
Statistic	*t* = −20.37, *p* < 0.001*	*t* = − 24.99, *p* < 0.001*	*t* = − 23.87, *p* < 0.001*	*t* = − 20.41, *p* < 0.001*
Fatigue				
Yes	62.18 ± 19.91	58.20 ± 19.06	55.55 ± 23.59	60.93 ± 20.29
No	80.31 ± 13.93	75.57 ± 12.11	78.64 ± 12.63	79.63 ± 13.38
Statistic	*t* = −12.50, *p* < 0.001*	*t* = − 12.59, *p* < 0.001*	*t* = − 13.62, *p* < 0.001*	*t* = − 12.70, *p* < 0.001*
Hair loss				
Yes	54.75 ± 27.84	56.96 ± 19.86	52.46 ± 27.06	54.29 ± 28.61
No	66.01 ± 18.17	60.91 ± 19.06	59.40 ± 23.06	64.72 ± 18.48
Statistic	*t* = −7.92, *p* < 0.00*	*t* = − 2.87, *p* < 0.004*	*t* = − 4.08, *p* < 0.001*	*t* = − 7.20, *p* < 0.001*
Bleeding				
Yes	48.53 ± 16.38	45.05 ± 12.56	39.11 ± 16.62	47.56 ± 17.44
No	76.18 ± 13.60	71.68 ± 14.98	72.70 ± 17.29	74.86 ± 13.79
Statistic	*t* = −37.05, *p* < 0.001*	*t* = − 37.83, *p* < 0.001*	*t* = − 39.27, *p* < 0.001*	*t* = − 35.14, *p* < 0.001*
Infection				
Yes	58.14 ± 19.09	53.47 ± 17.18	50.48 ± 22.05	57.05 ± 19.94
No	81.06 ± 11.77	78.57 ± 10.41	79.44 ± 12.89	79.66 ± 10.55
Statistic	*t* = −23.67, *p* < 0.001*	*t* = − 28.87, *p* < 0.001*	*t* = − 26.06, *p* < 0.001*	*t* = − 22.73, *p* < 0.001*

*Significant at *p* < 0.05

#### Psychological effects & self-esteem

The mean and SD of self-esteem for each domain of QOL of cancer patients was 24.59 ± 7.00. In correlation between psychological effects (anxiety and depression), self-esteem and domains of QOL of cancer patients, there was a positive and strong relationship between self-esteem and all domains of QOL. Also, those with depression and anxiety had lower scores on all domains of QOL (Table 4).

**Table 4:** Quality of life measures by psychological effects and self-esteem in cancer patients in Malaysia (*n* = 1620)

psychological effects	Physical health	Psychological health	Social relationships	Environment
	Mean ± SD	Mean ± SD	Mean ± SD	Mean ± SD
Anxiety				
Yes (GAD-7 ≥ 8)	62.86 ± 19.96	59.16 ± 19.38	56.67 ± 23.78	61.80 ± 20.40
No (GAD-7 < 8)	84.10 ± 10.00	75.44 ± 6.79	80.50 ± 6.11	81.53 ± 10.71
Statistic	*t* = −11.53, *p* < 0.001*	*t* = − 9.15, *p* < 0.001*	*t* = − 10.94, *p* < 0.001*	*t* = − 10.47, *p* < 0.001*
Depression				
Yes (PHQ-9 ≥ 10)	57.34 ± 18.91	52.38 ± 16.52	49.17 ± 21.45	56.14 ± 19.61
No (PHQ-9 < 10)	81.11 ± 11.32	79.20 ± 9.56	80.30 ± 11.53	80.08 ± 10.18
Statistics	*t* = −25.67, *p* < 0.001*	*t* = − 33.33, *p* < 0.001*	*t* = − 30.06, *p* < 0.001*	*t* = − 25.39, *p* < 0.001*
Self-esteem				
Mean ± SD	24.59 ± 7.00	24.59 ± 7.00	24.59 ± 7.00	24.59 ± 7.00
Statistics	*r* = 0.76, *p* < 0.001*	*r* = 0.78, *p* < 0.001*	*r* = 0.73, *p* < 0.001*	*r* = 0.74, *p* < 0.001*

*Significant at *p* < 0.05

#### Post-hoc comparison between overall quality of life with race, religion and type of cancer among participants

To provide specific information on which means were significantly different between QOL with race, religion and type of cancer, post-hoc comparison was conducted after Bonferroni adjustment with a significant level set at *p* = 0.005 level (2-tailed) (Table 5). Regarding race, the statistically significant differences were noted between Malay and Indian (*p* < 0.000), and Chinese and Indian (*p* < 0.000) races. In religion, the statistically significant differences were between Hindu and Islam (*p* = 0.000), and also between Buddha and Hindu (*p* = 0.000).

**Table 5:** Post-hoc comparison between quality of life with race, religion and type of cancer among participants

Characteristics		Mean differences (I-J)	*p*-value
Race (I)	Race (J)		
Malay	Chinese	- 0.83	1.000
	Indian	51.07	0.000*
	Other	33.15	0.613
China	Malay	0.83	1.000
	Indian	51.91	0.000*
	Others	33.98	0.584
Indian	Malay	- 51.07	0.000
	Chinese	- 51.91	0.000*
	Others	- 17.92	1.000
Others	Malay	- 33.15	0.613
	Chinese	- 33.98	0.584
	Indian	17.92	1.000
Religion	Religion		
Muslim	Buddhist	- 0.46	1.000
	Hindu	51.17	0.000*
	Christian	33.25	0.607
Buddhist	Muslim	0.46	1.000
	Hindu	51.64	0.000*
	Christian	33.72	0.600
Hindu	Muslim	- 51.17	0.000*
	Buddhist	- 51.64	0.000*
	Christian	- 17.92	1.000
Christian	Muslim	- 33.25	0.607
	Buddhist	- 33.72	0.600
	Hindu	17.92	1.000
Type of cancer (I)	Type of cancer (J)		
Breast	Ovarian	- 51.65	0.000*
	Cervical	- 18.39	0.169
	Colorectal	3.54	1.000
	Lymphoma	14.19	1.000
	Stomach	15.42	0.417
	Others	- 9.23	1.000
Ovarian	Breast	51.65	0.000*
	Cervical	33.26	0.06
	Colorectal	55.20	0.000*
	Lymphoma	65.85	0.000*
	Stomach	67.08	0.000*
	Others	42.42	0.005
Cervical	Breast	18.39	0.16
	Ovarian	- 33.26	0.06
	Colorectal	21.93	0.04
	Lymphoma	32.58	0.01
	Stomach	33.81	0.005
	Others	9.15	1.000
Colorectal	Breast	- 3.54	1.000
	Ovarian	- 55.20	0.000*
	Cervical	- 21.93	0.040
	Lymphoma	10.64	1.000
	Stomach	11.88	1.000
	Others	−12.78	1.000
Lymphoma	Breast	−14.19	1.000
	Ovarian	− 65.85	0.000*
	Cervical	−32.58	0.013
	Colorectal	−10.64	1.000
	Stomach	1.23	1.000
	Others	−23.42	0.214
Others	Breast	9.23	1.000
	Ovarian	−42.42	0.002
	Cervical	− 9.15	1.000
	Colorectal	12.78	1.000
	Lymphoma	23.42	0.214
	Stomach	24.66	0.050

*Significant at *p* < 0.005

Regarding the type of cancer, the actual differences between type of cancer and QOL was noted between breast and ovarian cancer (*p* < 0.000), colorectal and ovarian cancer (*p* < 0.000), lymphoma and ovarian cancer (*p* < 0.000) and ovarian and stomach cancer (*p* < 0.000). Regarding stage of cancer and QOL, the significant differences occurred between stage I and stage IV (*p* < 0.000).

#### Association between gender with type of cancer and treatment side-effects

Table 6 shows the association between gender with type of cancer and treatment side-effects. Based on the results, there was significant association between gender and treatment side effects (*p* < 0.001). The highest percentage of treatment side-effects were among females 888(96.3%) compared to males 625(89.6%). Among females and males who had treatment side-effects, a majority of them had more than three side-effects with 756(82%) and 519 (74.4%), respectively. There was also a significant relationship between type of cancer and gender (*p* < 0.001). Colorectal cancer was the highest cancer among males 436(62.5%) and the highest cancer among females was breast cancer 523 (56.8%).

**Table 6:** Association of type of cancer and treatment side-effect with gender among participants

Characteristics	Gender		Statistics	Eta square
	Male No. %	Female No. %		
Type of Cancer				
Breast	0(0.00%)	523(56.8%)	1206.17 *P* < 0.001*	0.86
Ovarian	0(0.00%)	62(6.7%)		
Cervical	0(0.00%)	143(15.5%)		
Colorectal	436(62.5%)	14(1.5%)		
Lymphoma	81(11.6%)	21(2.3%)		
Stomach	52(7.4%)	109(11.8%)		
Others	129(18.5%)	50(5.4%)		
Treatment side effect				
No side effect	73(10.5%)	34(3.7%)	Chi-square = 31.23 *P* < 0.001*	0.14
One side effect	30(4.3%)	32(3.5%)		
Two side effect	76(10.9%)	100(10.8%)		
≥ Three side effec	519(74.4%)	756(82.0%)		

*Significant at *p* < 0.005

#### Predictors of quality of life

To indicate determinant factor(s) of QOL, multiple regression analysis was performed. The assumption of linearity, homoscedasticity and normality of residuals were met. Based on the results; the main predictors for all domains of QOL among cancer patients in Malaysia were race, religious, cycle of cancer treatment, nausea, hair loss, bleeding and self-esteem. The strongest predictors of all domains of QOL in this study were nausea, hair loss and bleeding (refer to Table 7).

**Table 7:** Multivariate regression model for socio-demographic characteristics, clinical factor and physical side effect, psychological effects and self-esteem in cancer patients in Malaysia (*n* = 1620)

Characteristics	Physical health		Psychological health		Social relationships		Environment	
	B	p	B	p	B	p	B	p
Age								
< 45	−1.75	0.24	0.25	0.85	/^#^	/	0.47	0.77
45–54	0.37	0.71	6.21	0.00*	/	/	1.09	0.33
55–64	3.31	0.00*	0.97	0.18	/	/	1.65	0.06
> 65	Ref^+^		Ref		/	/	Ref	
Race								
Malay	Ref		Ref		Ref		Ref	
Chinese	5.36	0.64	− 0.11	0.99	− 1.18	0.30	5.47	0.65
Indian	2.28	0.00*	− 2.64	0.03*	− 3.72	0.01*	−6.73	0.00*
Other	−0.30	0.68	0.58	0.60	0.58	0.77	0.39	0.41
Religious								
Muslim	Ref		Ref		Ref		Ref	
Buddhist	− 8.17	0.47	−0.95	0.92	− 26.68	0.06	−9.56	0.43
Hindu	− 5.95	0.00*	− 2.31	0.01*	- 1.98	0.04*	− 7.62	0.00*
Christian	6.78	0.57	10.22	0.35	9.08	0.48	6.07	0.63
Marital status								
Married	/	/	/	/	/	/	Ref	
Single	/	/	/	/	/	/	−3.71	0.01
Divorce/Widow	/	/	/	/	/	/	−3.42	0.00*
Education level								
No formal education	−2.05	0.03*	0.12	0.89	/	/	− 1.81	0.08
Diploma & Less	Ref		Ref		/	/	Ref	
University level	−0.88	0.51	−2.22	0.07	/	/	−1.75	0.22
Working status								
Yes	Ref		Ref		Ref		Ref	
No	−1.04	0.26	−1.34	0.12	1.99	0.01*	−0.58	0.56
Retired	−2.68	0.00*	0.74	0.41	0.30	0.76	−1.89	0.07
Family history of cancer	/	/	1.78	0.00*	/	/	/	/
Type of Cancer								
Breast	Ref		Ref		Ref		Ref	
Ovarian	−4.89	0.00*	0.26	0.86	−2.71	0.17	−6.67	0.00*
Cervical	− 0.99	0.93	1.57	0.15	9.27	0.00*	0.88	0.48
Colorectal	−3.24	0.00*	0.30	0.70	0.74	0.45	−3.04	0.01*
Lymphoma	−2.08	0.13	−1.26	0.32	3.72	0.23	−2.38	0.11
Stomach	−8.57	0.00*	− 0.42	0.68	−8.43	0.00*	−8.19	0.00*
Others	−3.77	0.00*	−0.53	0.59	−7.79	0.00*	−3.37	0.03*
Cancer stage								
I	−0.53	0.69	0.54	0.66	−2.52	0.06	−1.57	0.28
II	−0.10	0.92	−0.10	0.91	3.62	0.00*	−0.46	0.69
III	0.68	0.34	−4.79	0.00*	0.73	0.39	0.26	0.73
IV	Ref		Ref		Ref		Ref	
Type of cancer treatment	−0.42	0.19	−1.83	0.00*	/	/	1.50	0.29
Cycle of cancer treatment	−3.88	0.00*	−1.57	*0.05*	7.14	0.00*	− 4.36	0.00*
Pain due to cancer (yes)	−4.58	0.00*	1.43	0.10	7.32	0.00*	− 5.57	0.00*
Vomiting (yes)	0.58	0.69	−5.59	0.00*	−5.39	0.00*	−0.61	0.69
Nausea (yes)	−9.51	0.00*	−10.90	0.00*	9.83	0.00*	−7.61	0.00*
Fatigue (yes)	−2.55	0.04*	− 0.44	0.70	−6.43	0.01*	− 3.97	0.00*
Hair loss (yes)	14.19	0.00*	5.42	0.00*	9.37	0.00*	13.35	0.00*
Bleeding (yes)	−7.89	0.00*	−6.74	0.00*	− 13.99	0.00*	− 6.84	0.00*
Anxiety (yes)	−5.35	0.00*	4.23	0.00*	0.17	0.92	−2.73	0.11
Depression (yes)	2.87	0.06	−0.42	0.77	−10.84	0.00*	1.41	0.39
Self-esteem	1.20	0.00*	0.90	0.00*	1.46	0.00*	1.25	0.00*
Constant	49.78	0.00	53.55	0.00	17.43	0.00	48.61	0.00
Adjusted R Square	0.69		0.70		0.68		0.65	
Model Fit Data	*F* = 112.42, *p* < 0.000		*F* = 113.24, *p* < 0.000		*F* = 128.28, *p* < 0.000		*F* = 88.39, *p* < 0.000	

^#^ The symbol of “/”means that variable not include in the final module of regression because significant in bivariate analysis; ^+^References Group; B, Unstandardized Coefficients; p, *significant at *p* < 0.05; © 1USD = 4 MY

These variables accounted for 69%, 70%, 68% and 65% of variability in physical health, psychological health, social relationships and environment domains of QOL of cancer patients, respectively.

Nausea, bleeding, hair loss were associated with lower scores in all domains of QOL. Also, cancer patients with anxiety had lower scores in all domains of QOL but were not significant for social relationships and environment. Compared with those working, retired patients had lower physical health score. Indian patients compared to Malay patients had lower scores on three domains of QOL included psychological health, social relationships and environment, but higher score on physical health. Self-esteem had positive association with all domains of QOL. Table 7 summarizes the results of the multiple regression analysis.

## Discussion

It is becoming increasingly difficult to ignore the fact that cancer patients with active disease and poor quality of life need more attention and supportive care. Generally, QOL is better without cancer but cancer is preventable by healthy lifestyle behavior such as smoking cessation, healthy diet, and regular physical activity and also early detection of cancer at an early stage, when it has a high potential for cure and used of less invasive treatment can improve the QOL of cancer patients [29, 30].

### Socio-demographic characteristics

The current study demonstrates that race, religion, and working status have significant effect on all domains of QOL of Malaysian cancer patients. Age and educational level also had significant effects on three QOL domains which included physical health, psychological health and environment. Religiousness such as seeking God’s love or protection, seeking help in religious literature and prayers was positively associated with better QOL and low level of psychological distress [31, 32]. The QOL was higher in the Buddha religion, most probably because Buddhist believe that birth, aging, illness, and death were the natural processes of life. Dealing with suffering is the common element of Buddhism [33]. Buddhist teachings have enduring relevance because they consistently relate death to life. It is usual to start dealing with death once one is faced with it. Buddhists believe one should begin a long time before that, so that pain and anxiety do not interfere with one’s ability to understand the situation [34]. However, the reason behind that does not explain why those with Buddha religion had higher QOL as compared to Hindu religion in our study; and needs further exploration. This might be due to unequal sample sizes of different groups being compared, as well as spiritual concerns among Indian patients which were associated with poor psychological health and poorer QOL [35]. This is supported by a previous study among an Indian population which found that 86% of patients with advanced cancers endorsed one or more spiritual concerns [35].

In addition, the consistent results of the present study in a Malaysian population with those in China and Western population support effect of age, working situations and religiosity on QOL of cancer survivors [5, 31, 36].

However marital status, gender and family history of cancer do not have any significant impact on every domain of QOL among cancer patients which is in line with a previous study conducted among 352 cancer patients in Ankara [37]. However, other studies like Miller et al. [38] or Bei Yan et al. [5] have found a relationship between age, education level and QOL which is in line with results of this study.

Based on the current study, all domains of QOL was higher among Chinese, followed by Malay and lowest among Indians. This may be due to socio-economic and also socio-cultural differences. There are some reasons for this circumstance. Firstly, Malay women place greater emphasis on beauty and reproductive health, and cancer treatment causes changes like hair loss, vomiting, nausea which may causes lower QOL as compared to Chinese women [39]. Secondly, in the Chinese culture, they believed the illness maybe a result of imbalance of cold and hot elements together with an obstructed flow of Ch’i [39], and the QOL is improved when there is a balance in all four elements. Consequently, when they were diagnosed with cancer, many Chinese place a greater emphasis on a balanced diet. Also, Chinese women do not fear the loss of their husbands, especially for those diagnosed with breast or gynaecology cancer as compared to Malay women [39]. Therefore Chinese women tend to place a greater emphasis on recuperating from their illness rather than worrying about body image and their marital relationship. The QOL (all domains) was lowest among the Indians as compared to the other races in Malaysia. Poor socio-economic status among the Indian population might be an important factor which could result in the lower QOL among Indians [40, 41]. This reason is supported by previous literatures which mentioned that a higher level of income has been linked to many aspects of better care of cancer patients such as rehabilitation and prompt treatment [5, 38].

### Clinical factors

Pain is one of the most important distressing symptoms of cancer which effect on all aspects of life [42]. The result of the current study conducted in Malaysia is in agreement with previous studies conducted in Iran [43], Germany [44] and Brazil [45], where patients with cancer pain reported poor QOL and higher levels of mood disturbance due to pain compared to patients without pain.

Previous work has identified that unsurprisingly, patients in the last stage of cancer have worse symptoms, function scores and poor QOL compared to the patients with early-stage cancer [46]. Our results supported that cancer patients with advanced cancer had greater deterioration and lower scores on all domains of QOL. This could be because patients with advanced cancer were more often bothered by problems of their physical and mental functioning. Currently, the palliative care developed in Malaysia, however still lack of this type of care in government hospitals in Malaysia.

The comparison of each domain of QOL in different cycles of cancer treatment, indicate that most of QOL aspects had progressive deterioration over the chemotherapy cycles. Those in 1st cycle of chemotherapy reported higher QOL in all domains as compared to those in the 2nd cycle of chemotherapy. This result is in line with studies done in German [47] and Iran [48]. A study conducted among 534 breast cancer patients in Seoul, Korea indicated that total score of QOL and each domains were higher among those not receiving chemotherapy [49]. Chemotherapy very often induces acute side-effects that usually develop at the end of the first cycle and beyond. When present, side-effects generally worsen QOL and the difference observed between cycles is not surprising.

Women diagnosed with breast cancer experienced very stressful life events such as insecurity, and feeling no longer desirable, with associated low body image and the added fear that their spouses might leave them for other women. All these would lead to a decrease in QOL [39, 50]. Similarly, a study conducted in Iran [51] found that Iranian women with breast and gynaecology cancer were solicitous that their husband married again. The common factor in both study is religions, as both groups are Muslim; where Muslim men can marry up to four wives [52]. In Malaysia, despite the growing burden of colon cancer the awareness of colorectal cancer screening among public for detecting colon cancer in the early stage very poor and also lack of information regarding this cancer for patients as compare to other cancer like breast and cervical cancer, which cause the lowest quality of life among colorectal cancer patients [40, 53].

### Treatment side effects

The results of the present study indicate that treatment side effect of cancer such as; vomiting, nausea, hair loss, anorexia, bleeding and infection are significantly associated with lower scores of all domain of QOL. Approximately 50% of patients with cancer will experience nausea and vomiting during the chemotherapy treatment; vomiting induced by chemotherapy may be prevented in around 70 to 80% of patients with the proper use of antiemetic agents, however, the control of nausea is more limited [54] The results are in line with those of several earlier studies reporting that vomiting, nausea and hair loss cause lower QOL among cancer patients [20, 55]. Gozzo et al. [56] similarly reported 93% of breast cancer patients who received chemotherapy had nausea and 87% of them had vomiting, this rate is higher than those found in the literature and had a negative impact on their QOL. Therefore, it is important to provide repetitive chemotherapy counselling so that pharmacists and clinicians are more aware of the side effects their patients are suffering from while receiving treatment. The findings of this study were found in line with previous studies which reported severity of nausea and vomiting of cancer patients were improved after providing chemotherapy counselling by pharmacists [20, 57]. Consequently, the need for the pharmacist involvement grew significantly with the shift from a disease-centered to a patient-centered care. With that shift, a patient’s quality of life became a measure that is, perhaps as important as the disease progression [58].

### Psychological effects (anxiety and depression)

One of our hypotheses in this study was that there is a significant association between anxiety and depression with all QOL aspects among cancer patients. In accordance with the present study, two other studies carried out in Germany [59] and Iran [43] showed that anxiety and depression were significantly correlated with impaired QOL. Findings of this and previous studies indicated that depression and anxiety are important psychological comorbidities of cancer patients which significantly alter the QOL of these patients [59]. Providing information about psychological effects of cancer to healthcare teams can assist in improving their treatment of cancer patients and subsequently improve the QOL of these patients. It has been proven that close relationships with the health care team, in spending more time with cancer patients has led to psychological improvement for cancer patients [60].

Self-esteem is related to the way people see themselves [61]. Our results highlighted there is a strong positive relationship between self-esteem and each domains of QOL. In this sense, Sidik et al. [62] have found that a great number of survivor cancer patients in Malaysia had feelings of isolation, anger and low levels of self-esteem after doing cancer treatment which indirectly affected their QOL.

### Predictors of QOL

The main predictors for all domains of QOL among cancer patients in Malaysia were race, religious, stage of cancer, cycle of cancer treatment, nausea, hair loss, bleeding and self-esteem. The strongest predictors of QOL in this study were nausea, hair loss and bleeding. Bleeding occurs in 6–10% of cancer patients which is distressing to patients and caregivers [63]. A longitudinal study conducted among 116 breast cancer patients for a duration of 2 years post treatment for breast cancer in San Francisco Bay Area, USA found that bleeding and fatigue were negative predictors for all domains of cancer patients QOL [64] which is in line with the results of this study.

Another predictor for this study was nausea. According to the cancer patients’ point of view, nausea is the most distressing side effect of cancer treatment which has negative impact on three domains of QOL including physical health, psychological health and environment health; and positively effect on social relationships domain [65]. Similarly, in a study conducted by Yost et al. [66] among 568 colorectal cancer patients with approximately 9 and 19 months post-diagnosis reported that nausea and vomiting were the strongest predictors for all domains of QOL.

People with high levels of self-esteem feel confident and capable of dealing with challenges and adapt themselves to different situations [67]. In this regard, when cancer patients have higher levels of self-esteem, it will be possible for them to see life in another way and this increases the level of QOL [61]. As found in this current study the increase in one score in self-esteem increases positively all domains of QOL (*p* < 0.00) which is in line with a study done by Bartoces et al. [68] among 145 cervical cancer survivors which reported self-esteem as the strongest predictor of health-related QOL [68].

Surprisingly in this current study, anxiety and depression were not the main predictors for all domains of QOL among cancer patients. This was probably due to other comorbidities which were not assessed in this study. Depression was the predictor for social relationship; however it was not a predictor for other domains. Anxiety was a predictor for two domains of QOL; which were physical and psychological health. In contrast to this result Tiara et al. [69] found that depression and anxiety to be extremely important factors for changing QOL among cancer patients after diagnosis or during the treatment. Therefore, it is recommended to provide educational programs based on the cognitive behavioral therapy and social and emotional support for improving QOL among cancer patients.

## Limitation and strength

There are several strengths for this study. A large sample size of this study provided sufficient statistical power to evaluate the impact of many factors which may had effect on QOL measures. Also, the validated Malay version of all instruments that were used in this study facilitated the detection of depression, anxiety, self-esteem and QOL as majority of participants were more comfortable in their national language. However, our study also has some limitations. We did not collect information on diabetes, other chronic disease, physical exercise, and social support. All of these factors are comorbidities which may have effect on QOL of cancer patients. Also, we do not have any information about QOL cancer patients before their diagnosis, thus we are unable to examine the change in QOL before and after cancer diagnosis. Finally, all information for this study were collected via self-reports with no objective measures to evaluate the cancer patients.

## Implication to practice

One of the most important concerns for cancer patients is quality of life and it can be used for assessing the QOL in oncology medicine [29]. This study shows the importance of measuring QOL and determining predictors of QOL among cancer patients. Finding of this study can be used as a fundamental research for developing further program to improve QOL among cancer patients. Also, Malaysia has a multi-ethnic population (60% Malay, 30% Chinese, and 10% Indian and other ethnic minorities) with different religions [70]. Based on the findings of this study, the information found regarding different socio-cultural beliefs and practices in Malaysia based on different races and religions could hopefully help oncology professionals in the planning of practical preventive strategies to greater patient comfort, satisfaction, improving QOL and enabling doctors to manage their patients better.

## Conclusion

In conclusion, it is important for health care workers especially pharmacists, oncologists, doctors and nurses to assist patients who are undergoing treatment for cancer, and create suitable strategies that meet psychological, clinical and physical needs of cancer patients with the aim to maintain and rehabilitate, and improve QOL of these patients. The findings of this study provide a scientific basis to develop a comprehensive program for improving the QOL of cancer patients in Malaysia.

## Abbreviations

CDR: Cytotoxic drug reconstitution
CTCAE: Common Terminology Criteria for Adverse Events
GAD-7: Generalized anxiety disorder-7
JKEUPM: Universiti Pura Malaysia Ethics Committee
MREC: Medical Ethics Committee
NMRR: National Medical Research Registry
PHQ-9: Patient Health Questionnaire-9
QOL: Quality of life
RAs: Research assistants
RSES: Rosenberg Self-Esteem Scale
SPSS: Statistical Package for Social Science
WHOQOL-BREF: WHO Quality of Life-BREF
